# Ocular Application of Oleuropein in Dry Eye Treatment: Formulation Studies and Biological Evaluation

**DOI:** 10.3390/ph14111151

**Published:** 2021-11-12

**Authors:** Susi Burgalassi, Erica Zucchetti, Elena Birindelli, Silvia Tampucci, Patrizia Chetoni, Daniela Monti

**Affiliations:** 1 Department of Pharmacy, University of Pisa, Via Bonanno 6, 56126 Pisa, Italy; susi.burgalassi@unipi.it (S.B.); erica.zucchetti@phd.unipi.it (E.Z.); silvia.tampucci@unipi.it (S.T.); patrizia.chetoni@unipi.it (P.C.); 2 Inter-University Center for the Promotion of the 3Rs Principles in Teaching & Research (Centro 3R), Largo Lazzarino 1, 56122 Pisa, Italy; 3 Fisiomed, Via Tosco Romagnola Ovest 210, Fornacette, 56012 Pisa, Italy; ebirindelli@gmail.com

**Keywords:** oleuropein, dry eye syndrome, liposomes, stability, hyperosmotic stress, oxidative stress

## Abstract

Background. Oleuropein is already known for its numerous pharmacological properties, but its activity in the ocular field has not yet been investigated. The study aims to verify a possible use of oleuropein (OLE)-based eye drops both in terms of efficacy in dry eye syndrome and stability in aqueous solution. Methods. OLE was co-precipitated with HP-β-cyclodextrin, and the obtained complex was encapsulated into liposomes prepared by hydration of a lipid film composed of Lipoid S100 and cholesterol with different pH buffer solutions. The hydrated vesicles were shrunk by ultrasonication or extrusion. The preparations were characterized from the physicochemical point of view by subjecting them to differential scanning calorimetry, ATR-FTIR, dynamic light scattering analysis, and microscopy. Subsequently, OLE protective activity against hyperosmotic and oxidative stress on rabbit corneal epithelial cells (RCE) was evaluated. Results. The liposomal vesicles obtained after extrusion showed a tendency towards greater encapsulation efficiency (up to 80.77%) compared to that obtained by sonication, and the liposomes hydrated in pH 5.5 solution tended to incapsulate more than the neutral ones. Ultrasonication produced two-dimensional populations of liposomes, the largest of which reached 2149 nm. On the contrary, the extruded liposomes showed homogeneous diameters of about 250 nm. Complexation with cyclodextrin and subsequent encapsulation in liposomes greatly increased the OLE stability in aqueous solution, especially at 4 °C and for the extruded formulations. OLE aqueous solution (OLE7.4-sol, reference) and neutral extruded liposomes (F7.4-e) were well tolerated on RCE cells. Moreover, OLE was able to control the effects of hyperosmolarity on ocular surface cells and to prevent oxidative stress-induced loss of cell viability.

## 1. Introduction

Oleuropein (OLE), belonging to a specific group of coumarin compounds, called secoiridoids, is the major phenolic constituent of Olea Europea, where it is mainly present in leaves but also in some part of fruit, of which it is responsible for the bitter taste. Scientific studies showed some pharmacological properties of oleuropein including anti-inflammatory, antioxidant, anti-cancer, hepatoprotective, neuroprotective, antiviral, and antimicrobial effects [1,2].

Taking advantage of these multiple properties, oleuropein could be used in the treatment of keratoconjunctivitis sicca, or dry eye syndrome (DES), that is a multifactorial disease of the tears and ocular surface with visual symptoms, eye disorders, and inflammation. In the world, people affected by DES are mainly older women in a range of about 5% to over 35% [3,4].

This pathology is characterized by lacrimal film hyperosmolarity that results from a tear flow reduction and/or increase in tear evaporation. Lacrimal hyperosmolarity is one of the central events in the vicious circle of DES and refers to the state in which osmolarity of tear exceeds that of the epithelial cell, leading to increased concentration of solutes and reduced cell volume. Tear hyperosmolarity causes epithelial damage by increasing the generation of reactive oxygen species (ROS) and activating an inflammatory cascade on the ocular surface with inflammatory mediators’ release and goblet cell apoptosis, which leads to a lower production of mucin and consequent lacrimal film instability. This aggravates tear hyperosmolarity, completing the vicious circle [4,5,6,7]. ROS may also be involved in DES, playing a role in various points of this cycle [8], and their overexpression on the ocular surface can be a consequence of an inadequate intake of antioxidant molecules due to the poor stability of the tear film [9].

DES is a chronic disease and requires long-term treatment [10] to improve patients’ conditions. Since tear hyperosmolarity seems to be of crucial importance in DES today, next to the most commonly used tear substitutes, osmoprotectants can be used to contain the damage to the ocular surface and break the vicious circle [11,12,13]. In addition, antioxidant agents, including those of natural origin, have been studied for controlling oxidative damages associated with DES [14,15].

The main scope of this study was the evaluation of cytotoxicity, protective activity from hyperosmotic stress, and antioxidant activity of oleuropein on rabbit corneal epithelial cells in order to verify a possible application of this compound in DES treatment. 

Problems linked to OLE are its sensitivity to light and to high temperature alongside the poor water stability [16] that makes it a critical compound to produce eye drops. 

Therefore, in the first step of this work, attention was focused on a method to improve oleuropein stability in aqueous solution. There are many strategies adopted to improve the natural compound’s stability, ranging from the simple addition of chelating and antioxidant agents to microencapsulation techniques [17] up to the most sophisticated nanotechnologies [18]. The encapsulation of the active ingredient into materials able to preserve the integrity of the molecule, such as polymers or lipids [19], can be exploitable. Novel nanostructured dosage forms such as nanoparticles, liposomes, niosomes, and nanomicelles offer a large number of advantages in overcoming limitations due to solubility, bioavailability, toxicity, and stability of natural products [18,20,21,22]. Moreover, a method employed to protect molecules from oxidation, light, and temperature degradation is the formation of a complex between the active ingredient and cyclodextrins [23]. 

On the basis of the literature data, in order to develop an oleuropein-based formulation for ocular application, the current work has focused on the combination of two different techniques: on the one hand, the complexation between OLE and hydroxypropyl-β-cyclodextrin by the co-precipitation method and, on the other hand, the encapsulation into a liposomal vesicular system composed by phospholipid Lipoid S100 and cholesterol.

## 2. Results and Discussion

### 2.1. Preparation and Physicochemical Characterization of OLE Formulation

The first objective of this work was to hinder the easy degradation of the natural active principle by starting from the complexation process of oleuropein with cyclodextrins. The product obtained was subjected to different analyses to demonstrate that the complexation had occurred.

The presence of interactions between oleuropein and HP-β-CD in the final complex (OLE/HP-β-CD co-precipitate) was investigated by differential scanning calorimetry (DSC) and ATR-FTIR analysis. 

**Figure 1:**
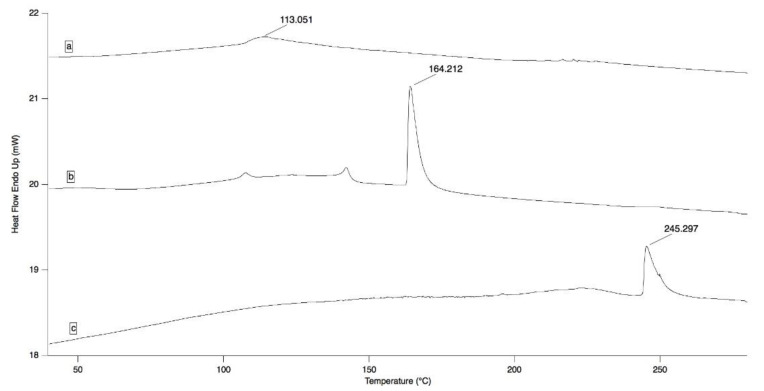
DSC thermograms of OLE (**a**), HP-β-CD (**b**), and OLE/HP-β-CD co-precipitate (**c**), at a scanning rate of 5 °C/min.

**Figure 2:**
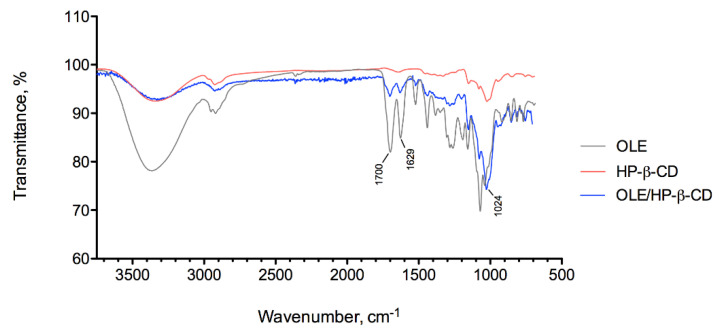
ATR-FTIR spectra of OLE, HP-β-CD, and OLE/HP-β-CD co-precipitate using a macro-ATR accessory with a diamond crystal.

DSC thermograms and ATR-FTIR spectra of the starting materials (OLE and HP-β-CD) and of OLE/HP-β-CD co-precipitate are illustrated in Figure 1 and Figure 2.

According to literature data [24,25], the DSC thermogram of pure OLE highlighted a broad endotherm around 100 °C (Figure 1a). Comparative thermograms showed the disappearance of the specific transitions of OLE and HP-β-CD from the OLE/HP-β-CD co-precipitate thermogram that gave way to an endothermic peak at 225 °C (Figure 1c); this may suggest interactions between the two materials, hinting the likely formation of the inclusion complex by the co-precipitation process.

By overlapping the ATR-FTIR spectra of the complex and the individual components (Figure 2), differences can be identified: the absorption bands at 1629 and 1700 cm^−1^, attributable to the C=O stretching of carbonyl and ester groups of OLE, respectively; decrease in intensity in OLE/HP-β-CD co-precipitate, as HP-β-CD does not show bands centered at these wavelengths; otherwise, the wide band in the 3600–3000 cm^−1^ spectral region, assigned to the O-H stretching of alcoholic and phenolic groups of oleuropein and cyclodextrin, and the band at 1024 cm^−1^, characteristic of carbohydrates, show a higher intensity of the signals due to the presence of the relevant groups in both compounds. Again, the changes in the spectrum of OLE/HP-β-CD co-precipitate indicate an interaction of the two materials that can be interpreted as the occurred complexation.

The choice of HP-β-CD and process to obtain the OLE inclusion complex was based on a series of assumptions: β-CD has a cavity size and a stable rigid structure that make it the ideal host for the inclusion of the most drugs, e.g., appropriate for aromatic rings [26], and proved to be more effective for OLE complexation than α- and γ-CD; the optimum stoichiometric ratio for complex formation was 1:1 with the maximum yield at neutral pH values [23,27]; the presence of the hydroxypropyl group increases its water solubility and decreases its cytotoxicity compared with the native CDs [28]. Furthermore, a monograph of HP-β-CD (hydroxypropyl-betadex) is available in the European Pharmacopoeia, and it has also been used in concentrations up to 12.5% in ophthalmic preparations without showing toxic or irritating effects on rabbit eyes [29,30,31].

The characterization of the inclusion complex was followed by its encapsulation in a nano-structured, vesicular system to improve the protection of the natural active and to give the formulation better characteristics for the delivery to the eye, also by selecting an appropriate method to allow a greater encapsulation and a longer stability of OLE.

Liposomes can be produced by many different techniques using different types of lipids. In this study, the standard hydration method for a phosphatidylcholine- and cholesterol-containing lipid film was used for preparing the liposomal dispersions subsequently sized by ultrasound or extrusion treatment. The liposomal formulations studied, named F7.4 or F5.5 depending on the pH value of the buffer solution used for the hydration, followed by -u or -e to indicate ultrasonication and extrusion as sizing method, are summarized in Table 1.

**Figure 3:**
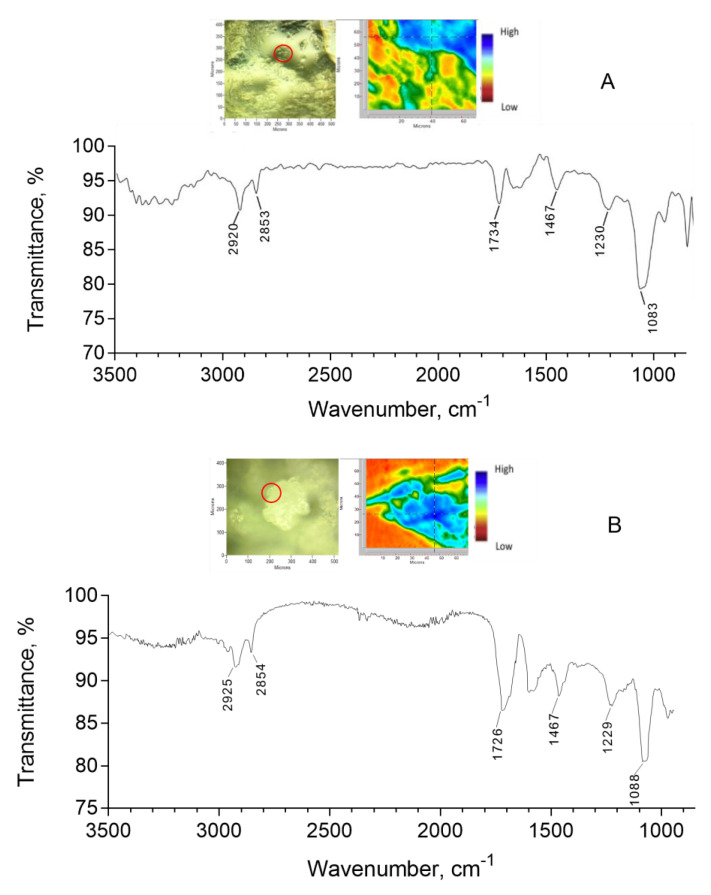
FTIR spectrum of F7.4-e (**A**) and F5.5-e (**B**) liposomal dispersions containing OLE/HP-β-CD co-precipitate dried in air using a micro-ATR accessory with a germanium crystal. The pictures show visible and FPA images of the compound: the infrared spectrum corresponds to the image pixel selected.

The FTIR spectra of the extruded liposomal dispersions (F7.4-e and F5.5-e) containing the OLE/HP-β-CD co-precipitate (Figure 3) show the characteristic bands at 2920 and 2850 cm^−1^ due to stretching of the aliphatic chains of the lipid components of the vesicles and, again, the wide band in the 3500–3100 cm^−1^ spectral region due to the O-H stretching of alcoholic and phenolic groups of oleuropein and cyclodextrin.

As published by Bonechi and colleagues [32], since the ATR-FTIR technique has a high sensitivity, these signals can be detected even if the complex is encapsulated in the aqueous core of the liposomal vesicles. These authors prepared liposomes containing tyrosol, hydroxytyrosol, and oleuropein and found the first two products, which were more water-soluble than OLE in the hydrophilic cavity of the vesicles, while oleuropein was detected within the phospholipidic bilayer. Other researchers developed oleuropein-loaded liposomes suggesting its encapsulation within the vesicular core [25]. In our case, the complexation with cyclodextrin made oleuropein more water-soluble as to justify its presence into the vesicles. It is known that the entrapment of water-soluble drug/cyclodextrin inclusion complexes into liposomes (DCL systems) would lead to the encapsulation of water-insoluble drugs in the aqueous core of vesicles [33], and this method has been largely used for increasing the encapsulation into aqueous core of liposomes of many hydrophobic drugs, such as riboflavin, ketoprofen, betamethasone, and curcumin [34,35,36,37,38].

The parameters of the physicochemical characterization of the prepared formulations are listed in Table 2.

The liposomal vesicles obtained after extrusion seem to show a trend to increase the encapsulation efficiency compared to that obtained by sonication, and the liposomes hydrated in acidic pH solution tend to encapsulate more than the neutral ones, although not with statistical differences.

The citrate buffer was selected for hydration of lipid film, as OLE shows higher stability in acidic aqueous solution [39], but also to increase the encapsulation of the OLE/HP-β-CD co-precipitate in the liposomal vesicles. Indeed, it is known in the literature that more effective encapsulation of cyclodextrins into the liposomes is achieved when the lipid film is hydrated with acidic citrate buffer (CBS) rather than when neutral phosphate buffer (PBS) is used, also reaching percentages of encapsulation over 95% [40]. In our study, the liposomal encapsulation in the acidic environment of the OLE/HP-β-CD co-precipitate results in an EE% that reaches 80.77 ± 1.35%.

Furthermore, in studies where OLE was encapsulated in a neutral environment, lower EE% values were measured. Nassir and colleagues [25] reported an EE% of 63.52 ± 4.15%, in the same range as that given by F7.4-u formulations (69.63 ± 1.02%), while Bonechi et al. found significantly lower values (30.2 ± 1.6%) [32].

Related to the methods for obtaining homogeneous dimensional populations, it can be noted that the ultrasonication produced two-dimensional populations of liposomes where the largest reached average dimensions of 2149 ± 388.9 nm. On the contrary, the extrusion process produces vesicles with more homogeneous diameters showing an average size around to 250 nm.

The size of the liposomal vesicles is an essential factor in ophthalmic administration where the application of liposomes containing formulations with greater dimensions can cause discomfort for the patient [41]. Moreover, in our research, smaller liposomal diameters are not reflected in decreased entrapment efficiency, as demonstrated in F5.5-e formulation where an EE% of 80.77 ± 1.35% in vesicles with size of 235.5 ± 14.94 nm was obtained.

The low polydispersity indexes of the extruded liposomal formulations indicate a mono-dispersion of the size of the vesicles, leading to the conclusion that, after extrusion, the liposomes remained adequately dispersed in the formulation, without giving rise to aggregation phenomena. Since the aggregation of the liposomal vesicles can be used as an index of the physical stability of the dispersion itself, from the data in our possession we can conclude that the liposomal dispersion containing OLE/HP-β-CD co-precipitate produces, from the physical point of view, a stable formulation.

**Figure 4:**
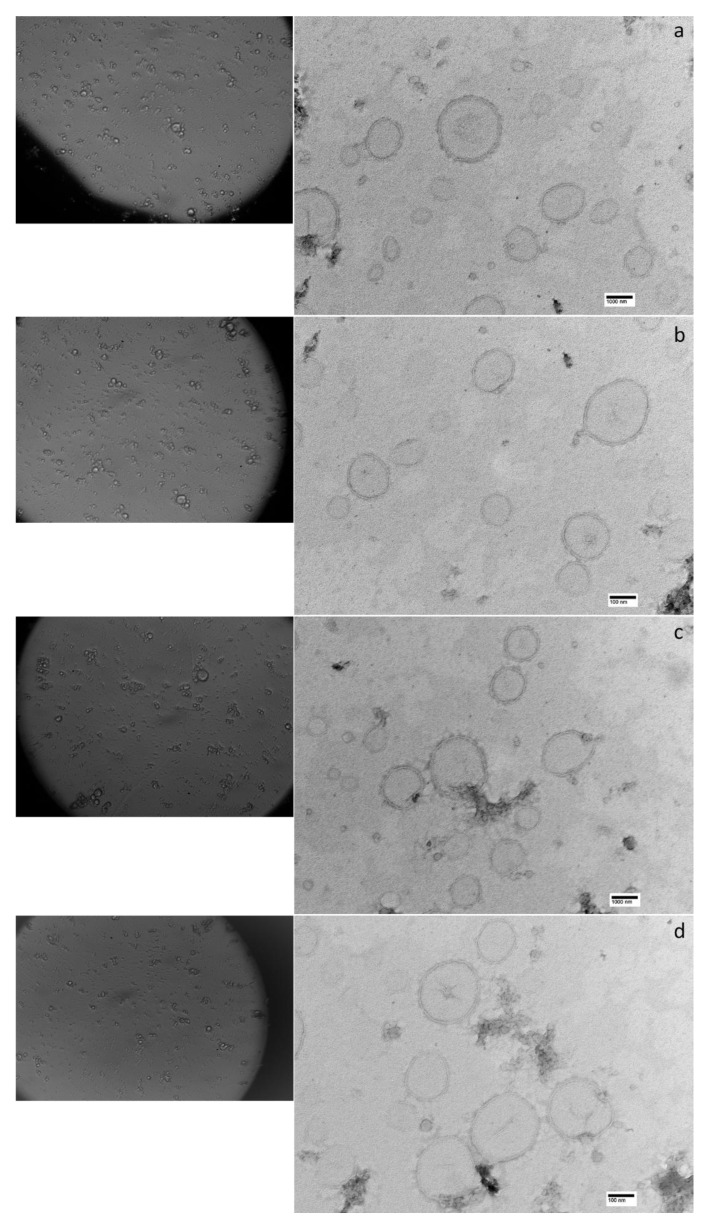
Photomicrographs of the liposomal dispersions by optical (on left) and TE microscopy (on right): (**a**) F7.4-u; (**b**) F7.4-e; (**c**) F5.5-u; (**d**) F5.5-e.

The non-aggregation of the liposomal vesicles is also evident from the photomicrographs obtained by optical and transmission electron microscopy (Figure 4). TEM microscopy has also allowed us to identify the structure of the liposomes obtained; in fact, the unilamellar nature was highlighted, as no concentric lipid bilayers could be identified. Furthermore, microphotographs were also useful for confirming the size of the liposomal vesicles prepared, although TEM typically measures mean sizes smaller than those determined by DLS. This trend is a consequence of the scattering of a small number of aggregated liposomes, which are also present at the high dilutions of the dispersion [42,43].

### 2.2. Stability Evaluation

The stability of the liposomal dispersions and OLE aqueous solutions in PBS (OLE7.4-sol) and CBS (OLE5.5-sol) was evaluated at room temperature (R.T.) and 4 °C away from light. The stability studies highlighted different degradation kinetics for the different storage conditions; all formulations showed a concentration exponential decay (first-order kinetics) when stored at 4 °C, while the degradation followed zero-order kinetics at 25 °C, except for OLE7.4-sol. The relevant results are listed in Table 3 as OLE half-life (t_50%_), the time required for the concentration to fall to half of its initial value.

Data show that OLE is more stable in weakly acidic than neutral solutions, both when stored at room temperature and at 4 °C, showing t_50%_ values of 79.47 and 96.61 days, respectively for R.T. and 4 °C for acidic solution, which dropped to 24.30 and 67.14 days, respectively, when the pH was neutral. It is noteworthy that OLE7.4-sol is much more stable when stored at 4 °C rather than at R.T., while the difference in storage temperature affects OLE stability to a lesser extent when in acidic solution.

OLE is described as stable in aqueous extract of olive leaves for about a week when stored at R.T., but it degrades completely in the following week [16]. Our results show a longer stability probably due to the fact that the studied aqueous solution contains OLE alone and no other extraction products of the plant; furthermore, the pH is controlled by a buffer system.

Moreover, it can be noted that the liposomal formulations demonstrate a higher stability of OLE when kept at 4 °C, both in neutral and acidic solutions. However, this greater stability is partly lost when the storage is carried out at R.T., especially for sonicated vesicles. In fact, the liposomes prepared by extrusion appear to protect OLE from degradation for longer times, mostly if refrigerated, highlighting differences related to the preparation methods of the liposomal formulations. To our knowledge, the literature does not report studies that generally correlate the stability of drugs encapsulated in liposomes with their preparation method. We speculated that this trend could be attributed to the larger size of the vesicles when dimensioned by ultrasound, resulting in a larger aqueous core that puts oleuropein more in contact with water and making it more subject to degradation.

### 2.3. Biological Assessment

The results obtained demonstrate that complexation with cyclodextrin and subsequent encapsulation in liposomal vesicles increase the OLE stability in aqueous solution, allowing its use in a formulation ready for ophthalmic administration, although it must be stored in the refrigerator.

Since the entrapment efficiency and stability were comparable in both the -e formulations, as well as the low polydispersity index, and since it could be prepared at physiological pH, the F7.4-e formulation was selected for a series of biological assessments to consider its use in restoring the ocular surface in cases of dry eye syndrome (DES).

**Figure 5:**
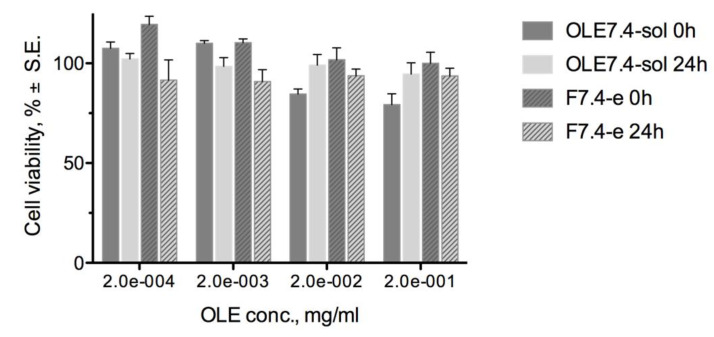
Cell viability of RCE cells after 60 min of contact with OLE formulations and subsequent recovery of 0 or 24 h in growth medium.

In the first instance, to evaluate the ocular surface tolerability, the F7.4-e formulation was subjected to cytotoxicity studies on an RCE (rabbit corneal epithelial) cell monolayer. OLE7.4-sol was used as reference. The results obtained are reported in Figure 5 as cell viability after 1 h of contact with OLE7.4-sol and F7.4-e formulations at increasing concentrations in growth medium and after a recovery time in growth medium of 0 and 24 h.

Oleuropein has proven to be well tolerated by RCE cells, at least at the tested concentrations, both for the solution and for the liposomal dispersion. Minimal cell viability values of 80% were observed at oleuropein concentrations up to 0.2 mg/mL, with no statistically significant difference between the data at 0 and 24 h of recovery, indicating that RCE cells do not require time to recover normal vitality after contact with oleuropein.

After checking the tolerability of oleuropein and its liposomal formulation, we moved on to evaluate the beneficial activities of the F7.4-e liposomal formulation on stressors related to the DES such as tear fluid hyperosmolarity and reactive oxygen species.

**Figure 6:**
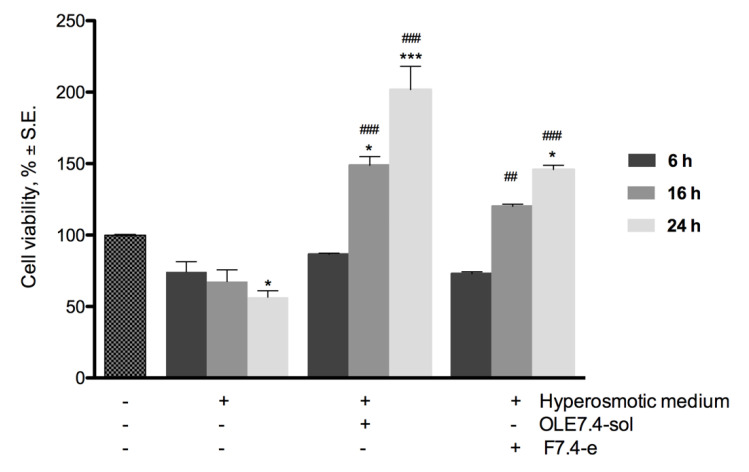
Cell viability of RCE cells pretreated 60 min with OLE formulations and subsequent addition and contact of hyperosmotic medium for 6, 16, and 24 h. * *p* < 0.05, ** *p* < 0.01, *** *p* < 0.001 were considered significant versus the control (untreated cells); # *p* < 0.05, ## *p* < 0.01, ### *p* < 0.001 were considered significant versus the cells in hyperosmotic medium for the same contact times.

In Figure 6, the results of the cell viability assay carried out on RCE after contact with a hyperosmotic medium are illustrated. The data show that 60 min oleuropein pre-treatment had a protective activity against hyperosmotic stress induced by saline solution mostly for a long time of contact. In fact, after 16 and 24 h of contact, cell viability values were statistically superior for the pre-treated cells, both with OLE solution and liposomes, in comparison with the not pre-treated and control cells.

Therefore, the effect of oleuropein seems to be time-dependent with a significant rise in cell viability with increasing contact time. After 16 h of contact with OLE in hyperosmotic medium, cell viability rose to 149 and 120%, respectively, for OLE7.4sol and F7.4-e, compared to 67.6% obtained for cells treated with only hyperosmotic medium. Values of 202 and 146% were reached after 24 h. These data highlight that the prolonged contact time with OLE seems to stimulate cell proliferation, leading to doubling the cell viability after 24 h of contact, although there are unfavorable hyperosmotic conditions.

Moreover, despite the encapsulation in liposomes, oleuropein maintains protective activity against hyperosmotic stress even if results attenuate with respect to OLE solution. This is probably due to a slower release of the active compound from the liposomal vesicles, as it is also complexed into cyclodextrin as well as many studies suggest for DCL systems [40].

Tear hyperosmolarity is believed to be the central event of inflammatory processes, leading to damaging the ocular surface and to triggering the onset of compensatory events in DES [44]. OLE, by controlling the effects of the hyperosmolarity on ocular surface cells, can improve dry eye symptoms and promote exit from the vicious circle of the syndrome.

Recent studies have demonstrated that oxidative stress damages the ocular surface cells and, together with the tear hyperosmolarity, is one of the contributing factors to DES [9].

**Figure 7:**
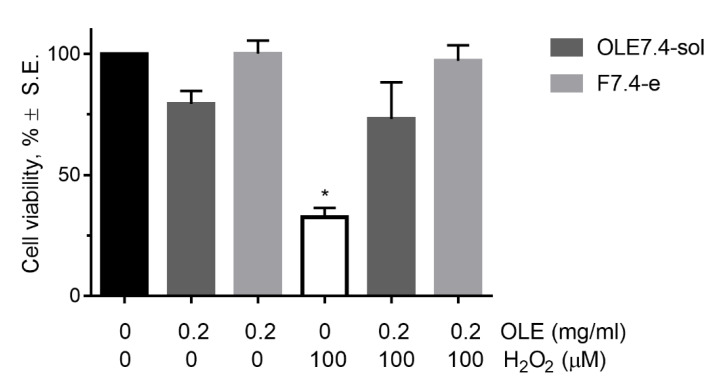
Cell viability of RCE cells after contact with OLE formulations and H_2_O_2_, alone (white bar) or after pre-treatment with OLE formulations. Black bar represents the untreated control. * *p* < 0.05 significant versus all other.

The results of the assay on the oxidative stress-induced damage indicate that pretreatment with 0.2 mg/mL OLE prevented H_2_O_2_-induced loss of cell viability, as shown in Figure 7 where RCE cell viability after the different experimental processes are reported. This preventive action is carried out both by the solution and by the liposomal formulation, to the same extent, as no statistically significant differences between cell viability values were observed. These data highlighted that OLE has a relevant antioxidant effect on corneal epithelial cells, and it is able to hinder oxidative stress-induced damages on the ocular surface.

Our results are consistent with those obtained by Shi and colleagues [45] on a human liver cell line, in which OLE exerted a protective action from H_2_O_2_-induced oxidative damage in concentrations ranging from 0.004 to 0.0160 mg/mL.

Oxidative stress-induced damages on the corneal surface have been investigated, and several clinical studies [46,47] highlighted a reduction in antioxidant enzymes in patients with DES, the extent of which was related to inflammation of the ocular surface and the severity of dry eye symptoms. Once again, it is shown that the intervention at a point of the vicious circle can result in an improvement in symptoms associated with the DES.

Taken together, the results of the biological assessment showed that OLE had a protective role against cell damage caused by several factors involved in DES, and its use in this disease could result in a benefit for patients.

## 3. Materials and Methods

### 3.1. Materials

The materials used in this study were oleuropein (OLE; Sigma-Aldrich, St. Louis, MO, USA); hydroxypropyl-β-cyclodextrin parenteral grade (HP-β-CD; Kleptose, Roquette Freres, Lestrem, France); phosphatidylcholine (Pho; Lipoid^®^ S 100; Lipoid, GmbH, Ludwigshafen, Germany); cholesterol (Chol; Sigma-Aldrich, St. Louis, MO, USA); Krebs–Ringer buffer solution (KRB, pH 7.4), variant without NaCl, with the following composition: 1.84 g/L D-glucose, 0.0468 g/L MgCl_2_, 0.34 g/L KCl, 0.1 g/L NaH_2_PO_4_, 0.18 g/L Na_2_HPO_4_; cell proliferation reagent WST-1 (Roche Diagnostic, Monza, Italy).

### 3.2. Cell Culture

The rabbit corneal epithelial cell line (RCE n. 95081046) was obtained from the European Collection of Authenticated Cell Cultures (ECACC, Salisbury, UK). The growth medium had the following composition: Dulbecco’s modified Eagle’s medium with Ham’s nutrient mixture F12 (1:1) (DMEM/F12) with addition of L-glutamine (2 mM), penicillin (100 UI/mL), streptomycin (0.1 mg/mL), amphotericin B (0.25 μg/mL), fetal bovine serum heat-inactivated (15% *v*/*v*) (Gibco, Rodano, I), insulin (5 μg/mL), and epidermal growth factor (10 μg/mL) (Sigma-Aldrich, St. Louis, MO, USA). Cells with passage numbers 10–15 were used. Cells were grown at 37 °C in a humidified atmosphere with 5% CO_2_.

### 3.3. Preparation of Formulations

#### 3.3.1. Complexation by Cyclodextrin

The oleuropein/HP-β-CD complex was formed by the co-precipitation method. Equimolar amounts of OLE (6.4 mg/mL) and HP-β-CD were dissolved separately into the same volume of acetone and acetone/water mixture (1:4 *v*/*v* ratio), respectively, mixed and continuously stirred for 24 h, and then evaporated under vacuum at 40 °C until complete drying. All operations were performed away from the light.

#### 3.3.2. Preparation of Liposomal Formulations

OLE liposomal formulations were prepared by conventional drug-lipid film hydration. A chloroform solution (20 mL) of Pho and Chol (135 and 7.63 mg, respectively; molar ratio 9:1) was dried to a thin film under reduced pressure at 35 °C in an evaporator rotating at 130 rpm (Rotavapor R-205, Buchi, Labortechnik AG, Flawil, Switzerland). The residual solvent was completely removed under reduced pressure overnight at room temperature. The resulting lipid film was hydrated in a rotary evaporator (95 rpm) for 4 h at 20 °C using 5 mL of either pH 7.4 phosphate (PBS) or pH 5.5 citrate (CBS) buffer solution containing an amount of OLE/HP-β-CD co-precipitate such to give a drug: lipid molar ratio of 1:30. To facilitate the detachment of the lipid film from the walls of the flask and the formation of more homogeneous liposomes, 20 glass spheres with a diameter of 3 mm were added.

The hydrated vesicles were shrunk applying two techniques: (i) by ultrasonication for 20 s at 22,000–23,000 Hz and 40 W (probe sonicator Microson XL 2000, Misonix, Farmingdale, NY, USA), maintaining the dispersion in an ice bath in order to avoid the fusion and/or sol-gel transition of the phospholipid membranes, breakdown of liposomes, and loss of the encapsulated drug; or (ii) by extrusion (Mini-Extruder, Avanti Polar Lipids Inc., Alabaster, AL, USA) through nitrocellulose filters: 21 passages through filter membranes with pores of 0.8 and 0.45 μm, and finally 7 passages through filter membranes with pores of 0.22 μm.

The liposomal dispersion containing OLE/HP-β-CD was undergone to ultrafiltration for removal of non-incapsulated drug by using VIVASPIN 6 filters (molecular weight cutoff 30 kDa, Sartorius, Firenze, Italy) centrifugated at 4000 rpm (centrifuge model PK120, ALC) at 20 °C for 3 h. The final pellet was resuspended in appropriate amounts of PBS or CBS to obtain an OLE concentration of 0.2 mg/mL.

### 3.4. Physicochemical Characterization

#### 3.4.1. Differential Scanning Calorimetry (DSC) Analysis

Differential scanning calorimetry measurements were carried on OLE/HP-β-CD co-precipitate and on the pure samples of OLE and HP-β-CD by a Pyris DSC 6 calorimeter (Perkin Elmer, Milano, Italy) in the 30–350 °C temperature range at a constant heating rate of 5 °C/min. Nitrogen, at the flow rate of 20 mL/min, was used as a purge gas throughout the analysis. Thermograms were recorded with Pyris Instrument Managing Software (Version 3.8, Perkin Elmer, Waltham, MA, USA) and processed by using IgorPro 6.05 (WaveMetrics Inc., Portland, OR, USA).

#### 3.4.2. ATR-FTIR Analysis and Focal Plane Array Imaging

ATR-FTIR spectra of OLE/HP-β-CD co-precipitate, of the pure samples of OLE and HP-β-CD, and of F7.4-e liposomal formulation were recorded with an IR Cary 660 FTIR spectrometer (Agilent Technologies, Santa Clara, CA, USA) using a macro-ATR accessory with a diamond crystal or a micro-ATR with a germanium crystal (liposomal formulation). The spectra were measured in a range from 4000 to 500 cm^−1^, with 32 or 64 scans both for background and samples. The liposomal formulation was dried in air before analysis to reduce interference due to the presence of water. Chemical imaging data were collected by Agilent’s ATR-imaging technique using an FTIR Cary 620 imaging system equipped with a 64 × 64 Focal Plane Array (FPA) detector cooled by liquid nitrogen. Background and sample spectra were measured from 3300 to 900 cm^−1^ with 256 scans.

#### 3.4.3. Dynamic Light Scattering Analysis

Size and size distribution of the liposomes were determined by measuring the rate of fluctuations in laser light intensity scattered by the liposomes immediately after their preparation by using a dynamic light scattering (DLS) Beckman Coulter^®^ N4 Plus (Beckman Coulter, Milano, Italy) and the CONTIN fit to treat non-monomodal distributions. The samples (about 15 μL) were diluted with ultrapure water (MilliQ, Millipore, Merck, Milano, Italy) previously filtered through a 0.45 μm RC membrane to an appropriate concentration chosen on the basis of the measurement intensity, which was in the range 5 × 10^4^ to 1 × 10^6^ counts per second (cps). The average size for each liposomal formulation was obtained on three different samples of formulations for which 3 runs were carried out, using an angle of 90° and run time of 200 s at 20 °C.

#### 3.4.4. Entrapment Efficiency

The entrapment efficiency, defined as the percentage of drug encapsulated in the lipid bilayers and/or aqueous compartments of the liposomal structures with respect to that initially added to the formulation, was determined by HPLC analysis after the following treatment: one volume of each liposomal formulation was mixed with ten volumes methanol, vortexed for 2 min, centrifuged at 13,000 rpm for 5 min (MicroCL 17, Thermo Electron, Rodano, Italy), and finally the supernatant was analyzed to determine the OLE amount. Each analysis was performed in triplicate.

The entrapment efficiency was calculated using the formula

*EE*% = *E_OLE_* * 100/*T_OLE_* (1)

where *E_OLE_* represents the amount of encapsulated drug and *T_OLE_* the total amount of drug initially added to the solution.

#### 3.4.5. Microscopy

The observation of liposomes was initially carried out by an optic microscope (MicroStar120, Reichert-Jung, Buffalo, NY, USA) at a magnification of 400×. After, TEM analysis was used to examine the ultrastructure of liposomes. Ten microliters of sample was allowed to adsorb for 3 min on a formvar/carbon coated copper grid (200 mesh). The grid was blotted with filter paper, washed with purified water, and subsequently negatively stained with 2% (*w*/*v*) aqueous solution of uranyl acetate, dried, and viewed under TEM at 80 kV (Jeol JEM 100SX, Jeol, Tokyo, Japan). The pictures were taken by a CCD camera (XR80B, AMT, Woburn, MA, USA).

### 3.5. Oleuropein Quantitative Analysis

The concentration of OLE in the liposomal formulations after lysis of the vesicles by methanol and in the aqueous solutions was determined by HPLC. The apparatus consisted of a LC-20 AT system with an UV SPD-10A detector and a CBM-20A interface (Shimadzu, Kyoto, Japan). The injection valve was a Rheodyne with a capacity of 20 µL, and a Lichrocart^®^ C18 (5 µm; 250 × 4.0 mm) column was employed. The mobile phase consisted of a mixture of water:acetonitrile:glacial acetic acid (70:29.9:0.1). The flux was 0.5 mL/min, the detection wavelength was 230 nm, and the retention time under these conditions was 8.0 min. The OLE amount in the samples was determined by comparison with external standard curves obtained by adding increasing amounts of the product to an appropriate solvent. The calibration curves were obtained by applying a least-squares linear regression analysis to experimental data using Prism software, version 8.0 (GraphPad Software Inc., San Diego, CA, USA) and were described by the following equations:

a. y = 27,000x − 1304; R^2^ = 0.9987, at a concentration ranging from 0.425 to 4.000 μg/mL in methanol (Limit Of Quantification = 0.093 μg/mL), to determine the entrapment efficiency;
b. y = 39,780x + 982; R^2^ = 0.9980, at a concentration ranging from 1.00 to 11.60 μg/mL in water (Limit Of Quantification = 0.322 μg/mL), for stability studies.

### 3.6. Stability Evaluation

The prepared liposomal dispersions were packaged in glass vials with a hermetic screw cap and stored in the refrigerator (4 °C) and at room temperature (about 20 °C), away from light. In the same conditions, solutions of OLE in pH 7.4 phosphate (PBS) and in pH 5.5 citrate (CBS) buffers were also stored. At predetermined time intervals, aliquots of the dispersions were taken and analyzed for the quantitative determination of residual OLE after addition of methanol and vortexing to lyse the lipid vesicles, as already described in Section 3.4.4.

In the stability study, the times in which the OLE concentration was reduced by 50% (t_50%_) were calculated from the equation that best described the curve of experimental data when OLE residual percentage versus time was plotted by using Prism software, version 8.0 (GraphPad Software Inc., San Diego, CA, USA).

### 3.7. Biological Assessment

#### 3.7.1. Cytotoxicity Studies

The determination of the toxicity level of OLE on the rabbit corneal epithelial cell line (RCE) was performed by a colorimetric method using the cell proliferation reagent WST-1. This method allows one to estimate the number of viable cells present in culture and, thus, to evaluate the effect of the treatment with a potential toxic agent on the viability of the cellular population.

The assay is based on cleavage of the tetrazolium salt WST-1 by mitochondrial enzymes to produce formazan salt, completely soluble in water and with cherry red coloration. Only viable cells are able to reduce WST-1, whose staining is therefore proportional to viable cell number.

The RCE cells were plated in 96-well plates at a concentration of 3 × 10^4^ cells/well. After 24 h, at approximately 70% confluence, the medium was completely aspirated, and cells were treated with 100 μL of OLE solution for 60 min. Subsequently, the reaction medium was aspirated, the cells were washed twice with DMEM/F12, and 100 μL of fresh growth medium was added. Immediately after, or after a 24 h recovery time, 10 μL of WST-1 was added, the cells were incubated for 2 h at 37 °C in a humidified atmosphere with 5% CO_2_, the microplate was thoroughly shaken for 1 min, and finally the absorbance was determined at 450 nm using a microtiter reader (Asys UVM 340; Biochrom, Cambridge, UK). The background absorbance was measured on wells containing only the dye solution and the culture medium.

The results were expressed as percentage of the absorbance of treated versus no-treated wells (control).

#### 3.7.2. Evaluation of the Protective Activity against Hyperosmotic Stress

The RCE cells were plated in 96-well plates at a concentration of 3 × 10^4^ cells/well. After 24 h, at approximately 70% confluence, the growth medium was aspirated and replaced with 50 μL of test solutions, all containing 0.2 mg/mL of OLE. After 60 min exposure, 100 μL of hyperosmotic medium (NaCl in growth medium, 487 mOsmol/kg) was added, and the plates were incubated for 6, 16, or 24 h. The final osmolarity of the treatment medium was about 440 mOsmol/kg as a result of the dilution of the hyperosmotic solution by the test solutions. Subsequently, the reaction medium was aspirated, the cells were washed twice with DMEM/F12, 100 μL of fresh growth medium, and 10 μL of WST-1 was added to each well. After incubation for 2 h at 37 °C in a humidified atmosphere with 5% CO_2_, the microplate was thoroughly shaken for 1 min, and finally absorbance was determined at 450 nm using a microtiter reader (Asys UVM 340; Biochrom, Cambridge, UK). The background absorbance was measured on wells containing only the dye solution and the culture medium.

The results were expressed as percentage of the absorbance of treated versus no-treated wells (control) and wells with only hyperosmotic medium.

#### 3.7.3. Evaluation of Antioxidant Activity

The RCE cells were plated in 96-well plates at a concentration of 3 × 10^4^ cells/well. After 24 h, at approximately 70% confluence, the medium was aspirated, and the cells were treated for 30 min with 50 μL of test solution containing 0.2 mg/mL of OLE in growth medium. After that, 100 μL of 100 μM H_2_O_2_ solution was added, and the plates were incubated for 4 h. Subsequently, after aspiration of the reaction medium and washing twice with DMEM/F12, 100 μL of fresh growth medium and 10 μL of WST-1 were added in each well. Finally, the cells were incubated for 2 h at 37 °C in a humidified atmosphere with 5% CO_2_, then cell viability was evaluated as described in the previous paragraphs.

### 3.8. Statistical Analysis

Data related to size distribution were reported as mean ± standard error (S.E.) of three different samples of formulation that underwent three runs each.

Data related to in vitro cell viability were reported as mean ± S.E. of at least three independent experiments, each performed in triplicate.

Statistical significance between two groups was analyzed by Student’s *t*-test, while one-way analysis of variance (ANOVA), followed by Tukey’s post hoc test, was used for multiple comparisons. At least a *p*-value < 0.05 was considered statistically significant.

All data processing was performed using Prism software, version 8.0 (GraphPad Software Inc., San Diego, CA, USA).

## 4. Conclusions

Recently, drug-in-cyclodextrin-in-liposome (DCL) systems have been investigated by several authors as a new approach that combines the advantages of using cyclodextrins and liposomes [33,35,48,49,50,51] in the formulation at the same time. Of all the options, DCL preparation is becoming a preferred choice for the delivery of the lipophilic, photolabile, and hydrolysis-sensitive drugs.

To our knowledge, this is the first report demonstrating the activity of DCL preparations in protecting OLE from degradation as well as their use in the ophthalmic field.

Although there is much literature on the use of natural products to relieve dry eye symptoms, OLE has never been employed for this purpose. One of the reasons could be related to its poor stability in aqueous solution. The improvement of this aspect might make OLE attractive as an active agent for ophthalmic use.

The activities showed by OLE liposomal formulation on corneal epithelial cells encourage the continuation of studies on oleuropein as a possible supplement for eye wellness, taking into account that the weak decrease in the activity of the formulation compared to the solution is balanced by the greater stability over time. Further research and studies will necessarily concern the in vivo evaluation of the ocular tolerability and activity on dry eye animal models to definitively verify the use of OLE in management of DES.

## Figures and Tables

**Table 1:** Liposomal formulations under study.

Label	Hydration Buffer	Sizing Method
F7.4-u	PBS ^1^	Ultrasonication
F7.4-e	PBS	Extrusion
F5.5-u	CBS ^2^	Ultrasonication
F5.5-e	CBS	Extrusion

^1^ PBS = pH 7.4 phosphate buffer solution; ^2^ CBS = pH 5.5 citrate buffer solution.

**Table 2:** Physicochemical characteristics of the liposomal dispersions under study.

Formulation	EE % (±S.E.)	Size (nm ± S.E.)	Polydispersity Index
F7.4-u	69.63 ± 1.02	240.9 ± 53.64 1863 ± 246.0	
F7.4-e	77.64 ± 3.08	268.2 ± 6.53	0.362 ± 0.0268
F5.5-u	72.98 ± 5.62	242.4 ± 38.19 2149 ± 388.9	
F5.5-e	80.77 ± 1.35	235.5 ± 14.94	0.310 ± 0.0317

**Table 3:** Stability of the formulations under study, t_50%_ (days): in brackets the determination coefficient of the curve or straight line that best fits the degradation kinetics.

Formulation	t_50%_ (days)	
	4 °C	R.T.
OLE7.4-sol	67.14 (0.958)	24.30 (0.998)
OLE5.5-sol	96.61 (0.984)	79.47 (0.871)
F7.4-u	89.68 (0.991)	50.82 (0.985)
F7.4-e	156.1 (0.995)	88.45 (0.936)
F5.5-u	90.93 (0.991)	50.90 (0.869)
F5.5-e	141.8 (0.946)	88.42 (0.995)
